# Perforator-based flaps for the treatment of burn scar contractures: a review

**DOI:** 10.1186/s41038-017-0071-2

**Published:** 2017-02-27

**Authors:** C. M. Stekelenburg, R. E. Marck, P. D. H. M. Verhaegen, K. W. Marck, P. P. M. van Zuijlen

**Affiliations:** 1 Burn Center and Department of Plastic, Reconstructive, and Hand Surgery, Red Cross Hospital, Vondellaan 13, 1942 LE Beverwijk, The Netherlands; 2 Department of Plastic, Reconstructive, and Hand Surgery, University Medical Center, Utrecht, The Netherlands; 3 Department of Plastic, Reconstructive, and Hand Surgery, Academical Medical Center, Amsterdam, The Netherlands; 4 Department of Plastic, Reconstructive, and Hand Surgery, MOVE Research Institute, VU University Medical Center, Amsterdam, The Netherlands; 5 Department of Plastic, Reconstructive, and Hand Surgery, Isala, Zwolle, The Netherlands; 6 Department of Plastic Surgery, Medisch Centrum Leeuwarden, Leeuwarden, The Netherlands

**Keywords:** Burn, Scar, Contracture, Perforator flap

## Abstract

Patients with burn scars often experience functional problems because of scar contractures. Surgical treatment may be indicated for those burn scar contractures. If the contractures are small and linear, the contraction bands can be treated with local transposition flaps like the Z-plasty. Broader, diffuse contractures are more challenging and require a different surgical approach, such as the use of local tissue. The use of perforator-based flaps is promising; however, their true clinical significance for this type of burn reconstructions still needs to be determined. Therefore, we performed a review to evaluate the role of perforator-based flaps for burn scar contracture treatment. Electronic databases were searched using a predefined search strategy. Studies evaluating the long-term outcome of perforator-based flaps for the treatment of burn scar contractures were included. The methodological quality was tested and data was summarized. Five hundred and ten papers were identified of which eleven met the inclusion criteria. One study was a randomized controlled trial; ten were cohort studies of a pre-postoperative design. The papers described outcomes of free flaps and local flaps. Most studies had methodological shortcomings and used inappropriate statistical methods. Perforator-based interposition flaps appear to be highly relevant for burn scar contracture treatment. However, due to the paucity and low quality of the studies that were assessed, no definitive conclusions about the true clinical significance could be reached. And therefore, only recommendations could be given for improvement of the quality of further primary research on the effectiveness of perforator-based flaps for burn scar contracture release.

**Electronic supplementary material:**

The online version of this article (doi:10.1186/s41038-017-0071-2) contains supplementary material, which is available to authorized users.

## Background

Today, most patients will survive severe burn injuries, but they will not escape the burden of severe scarring. The functional limitations of scarring mainly result from contraction of the scarred tissue that will leave contractures at joints. Surgical treatment is often indicated for those burn scar contractures. If the contractures are small and linear, the contraction bands can be treated with local transposition flaps like the Z-plasty. Broader, more diffuse contractures are more challenging to the reconstructive surgeon and require a different surgical approach. First, the tension of the contracted scar has to be released, which results in a considerable defect. Subsequently, this defect needs to be reconstructed. Closure of this defect can be obtained by split-skin and full-thickness skin grafts, but skin grafts have the disadvantages of an uncertain ingrowth in the recipient area and a certain recurrence of contraction [1, 2]. For these reasons, flaps should be preferred because they provide healthy skin and subcutaneous tissue that serves as a gliding plane. Moreover, flaps maintain their original blood supply. Although blood supply is essential for the survival of flaps, flaps can be raised without the knowledge of its feeding vessels, the so-called random flaps. These flaps have limitations in width and length because of their random blood supply. Preferably, skin flaps should therefore be designed according to their specific blood supply. If the vascularization is improved, larger flaps can be raised with larger arcs of rotation.

The discovery and better utilization of the feeding vessels of the skin, the perforators, therefore caused a breakthrough in battle between blood supply and survival of the flap. Taylor et al. performed much basic research on these vessels and their role in vascularization of the skin [3]. Because of the knowledge on perforators, we can harvest many types of skin flaps as long as it incorporates a perforator bundle of an artery and vein. The introduction of these so-called perforator flaps was therefore a milestone in reconstructive surgery and has significantly enlarged the possibilities for all sorts of oncological and traumatic defects both as free and local flaps. Perforator-based flaps are considered to be a breakthrough for burn scar reconstruction as well.

Perforators are vessels that perforate the fascia underneath the subcutis and run almost in a straight line through the subcutis to the skin. So if the location and course of perforators are considered in the flap design, the safety of the flap might be improved. The body contains a few hundred of such perforating vessels with a diameter of more than 0.5 mm [3]. Studies were published on the use of a perforator to increase the flap versatility and viability. Niranjan et al. performed a lot of clinically related research on the usage of perforators for indications such as large defects following excision of malignant skin lesions and selected trauma cases [4], but perforator-based flaps are also useful in reconstructive surgery as local flaps: the group of Hyakusoku and Ogawa has been working on the propeller flap techniques since 1991 [5, 6].

Understanding vascularization of tissue by perforators and their feeding larger (axial) vessels formed the basis for microsurgery, free flaps, and different types of local flaps.

Challenged by the many publications on perforator flaps and our own experiences with them, particularly in burn contracture surgery, we foresee that the role of perforator-based flap surgery in diffuse burn scar contracture will increase significantly in the next future. According to the available literature and information in textbooks, the true clinical significance of these flaps for this type of burn reconstructions still needs to be determined. Therefore, we performed a review to evaluate the role of perforator flaps for burn scar contracture treatment.

## Review

### Methods

#### Types of studies

Studies that evaluated the effect of perforator-based techniques for burn scar contracture reconstruction were included. Because a low number of randomized controlled trials (RCTs) was anticipated, not only RCTs were included but also single-intervention studies (pre-post comparison) and cohort studies.

#### Types of participants

All studies concerning reconstructive procedures for burn scar contractures by means of any type of perforator-based flap were included.

#### Types of interventions

Studies examining the effect of any type of perforator-based reconstruction technique after burn scar contracture release were included. If a comparator intervention was described, it could be any other reconstruction technique, placebo intervention, or no intervention.

#### Types of outcome measures

Since we were interested in sustainable functional results, we included studies that described the long-term outcomes, preferably with a specified follow-up period of at least 2 months. Typical outcome measures were functional improvement, the range of motion (ROM), planimetry (surface area measurements), and scar quality.

#### Other aspects of eligibility

Studies were excluded from the analysis if they focused on other surgery than burn scar contractures. To avoid inclusion of small, possibly selective patient series, only studies with an inclusion number of at least 15 procedures were included.

#### Search methods for identification of studies

We conducted a search in the PubMed, EMBASE, and Cochrane databases, from 1960 to November 1, 2016. The following search strategy was used for the PubMed search:

- Mesh Cicatrix, Scar* OR Burn*
- Contracture* OR scar*
- MesH descriptor Reconstructive Surgical Procedures, Reconstruct*
- MesH descriptor Surgical Flaps (explode all trees), Perforator

Furthermore, we performed a search at the WHO Clinical Trials Registry Platform Search Portal (www.who.int/trialsearch) and www.clinicaltrials.gov for studies.

#### Data collection and analysis

Three authors (CS, RM, and PvZ) independently screened the titles, abstracts, and text of the obtained manuscripts. The Newcastle-Ottawa quality assessment scale was used to assess the quality of the included studies (Additional file 1: Figure S1) [7]. Studies were judged on three broad perspectives: selection, comparability, and outcome. Discrepancies between the review authors were resolved by discussion, if necessary.

#### Statistical analysis

Statistical pooling of the data per outcome would only be undertaken for studies that are comparable (concerning study design, type of intervention, outcome description, and statistical analysis) and that present sufficient data to perform pooling.

### Result

#### Search results

After de-duplication, 510 references were identified from the before mentioned electronic databases. After screening the titles and abstracts, 409 studies were excluded because they were not on the treatment of burn scar contractures, they were not written in the English language, or there was no abstract available. Finally, 11 studies were retrieved for full evaluation. The other 90 studies were not eligible because it often concerned (1) a narrative description instead of a trial discussing the outcome of a certain technique and (2) the outcome description was insufficient.

### Main description of studies

One RCT was identified [8]. This trial compared the effectiveness of perforator-based interposition flaps versus full-thickness skin grafts (FTSGs) for the treatment of burn scar contractures (this paper was “in press” at the moment that the manuscript was prepared but it was not excluded from the analysis). The other ten papers were cohort studies with a pre-postoperative design: patients were measured before surgery and at a defined time period postoperatively. Most studies described a single procedure [9–15], one study described the outcomes of different adaptations of one flap [16], and one study compared the outcome of different flaps [17]. The range of follow-up periods varied from 2 months [18] to 8 years [11]. Overall, the sample size studied in the different papers was small ranging from 15 to 42.

### Outcome measures

The two most used outcome measures in the included studies were ROM [15, 17, 19] and surface area measurements of the flaps [8, 12, 14]. Range of motion was measured in degrees in the area of interest, which was either the neck or shoulder in most studies. Some authors measured the ROM preoperatively and compared this to the ROM postoperatively [8, 9]. Others compared the postoperative ROM to the normal or “ideal” ROM [10, 17]. Four studies only provided a narrative description of the long-term outcome, without presenting data [11, 13, 16, 18]. Flap area measurement was performed by measuring the width of the flaps [8, 12, 14] or the surface area [8, 14].

### Effect of interventions

The effectiveness of the interventions is summarized in Table 1. The different flaps that are described in the included papers can be divided in two groups: free flaps [17] and local flaps [8–18]. Tsai et al. mainly investigated the effectiveness of the anterior lateral thigh flap and observed a significant increase in ROM of the neck (Table 1). The other included papers studied the use of different local flaps. Of these studies, seven studies described the use of local flaps that are based on a specific [7] perforator that is part of the nomenclature, such as the (pre-expanded) thoracodorsal perforator flap [9, 15], the cervical artery flap [10, 16], the occipito-cervico-dorsal flap [11], and the supra clavicular artery flap [12]. In only three studies [9, 10, 15], the long-term ROM was measured (in degrees) and showed either an increase in ROM or a postoperative ROM that did not significantly differ from a normal ROM.

**Table 1:** Summary of outcome in included studies

Study	Mean follow-up (range)	Short-term follow-up	Long-term follow-up				Statistical analysis
		Necrosis flap (%)	ROM measurement	Increase in ROM (preop → postop)	Increase in flap size	Outcome measures validated?	
Ogawa [11]	5 years (1–8)	5/32 (16%) partial	–	–	–	–	No
Er [9]	Not mentioned	0/15 (0%)	Shoulder abduction	112° (47 → 159)	–	Goniometry: retrospective chart review	No
Tsai [17]	11 months	0/40 (0%)	Neck extension	24° (92 → 116)	15–38% ↑ flap width at 11 months	Geometry/planimetry	No
			Lateral flexion	10° (26 → 36)			
Ogawa [16]	Not mentioned	4/41 (10%) partial	–	–	–	–	No
		1/41 (2%) complete					
Rashid [12]	22 months (12–48)	2/27 (7%) epidermiolysis	–	–	63% ↑ flap width at 1 year	Geometry/planimetry	No
Waterston [18]	9 months (2–60)	1/23 (4%) complete	–	–	Stretching of flap was observed	–	No
Verhaegen [14]	8 months (3–14)	4/22 (18%) partial	–	–	16% ↑ flap surface area at 8 months	Planimetry	Yes
Tucker [13]	Not mentioned	3/22 (14%) partial	–	–	–	–	No
Wang [15]	Not mentioned (range 1–5 years)	0/15 (0%)	Neck extension and lateral flexion compared to normal ROM	Postoperative extension and lateral flexion not significantly differing from normal ROM	–	Flap geometry, measurement of cervical motion (Photo Measures software)	Yes
Li [10]	>1 year	1/15 (7%) partial	Neck extension	Neck extension >110° in all patients	–	Goniometry	No

*ROM*: range of motion

Finally, four studies investigated the effectiveness of local flaps based on “ad hoc” perforators (sometimes also referred to as free-style perforator flaps) [8, 13, 14, 18]. In the RCT, a significant difference in surface area and width between flaps and FTSGs was documented after 1 year, in favor of the flaps. This was in line with the findings in the study of Verhaegen et al., which showed an increase in width and surface area of the perforator-based flaps 7 months postoperatively. The other two studies only used a narrative description of the long-term outcome [13, 18].

### Quality assessment

The results on the quality assessment are summarized in Table 2. The quality of most of the included studies was low; especially outcome parameters for long-term follow-up were insufficiently described. Only the RCT scored maximal on the items selection, comparability, and outcome [8]. Because only one RCT was included, the same quality assessment scale (the Newcastle-Ottawa quality assessment scale) was used for all the included studies and the results were presented together in Table 2.

**Table 2:** Quality assessment using the New Castle-Ottowa scale

Study	Year	Study type	Selection				Comparability	Outcome		
			Representativeness of the exposed cohort	Selection of the non-exprosed cohort	Ascertainment of exposure	Outcome of interest not present at start	Comparability of the cohorts	Assessment of outcome	Follow-up long enough	Adequacy of follow-up of cohorts
Ogawa [11]	2004	Cohort—retrospective	*						*	
Er [9]	2005	Cohort—pre-postoperative design	*			*				
Tsai [17]	2006	Cohort—pre-postoperative design	*		*	*		*	*	
Ogawa [16]	2006	Cohort—retrospective	*						*	
Rashid [12]	2006	Cohort—pre-postoperative design	*			*			*	*
Waterson [18]	2008	Cohort—retrospective	*						*	
Verhaegen [14]	2011	Cohort—pre-postoperative design	*		*	*		*	*	
Tucker [13]	2011	Cohort—retrospective	*							
Wang [15]	2014	Cohort—pre-postoperative design	*					*	*	*
Li [10]	2015	Cohort—retrospective	*			*		*	*	*
Stekelenburg [8]	2017	RCT	*	*	*	*	*	*	*	*

A star (*) indicates that a measure was adequately addressed in this study. A study could be awarded a maximum of one star for each numbered item within the Selection and Exposure categories. A maximum of two stars could be given for Comparability.

### Statistical analysis

Statistical pooling could not be undertaken because the included studies were too heterogenic in terms of study design, type of intervention, outcome description, and statistical analysis. Therefore, performing a meta-analysis was not possible to perform. Although ten studies were of a comparable design (a pre-postoperative cohort study), they varied in the type of outcome measure that was used. Papers that used the same outcome description (ROM) did not consistently measure the same location (shoulder versus neck) or the same movement (i.d. extension or lateral flexion).

### Discussion

This review aimed to provide more scientific deepening to our knowledge of the outcome of perforator-based procedures for burn scar contractures. The raw conclusion of this review is that a sound scientific underpinning of the perforator-based procedures for burn contracture reconstructions is lacking so far. The total number of clinically interesting publications contrasts sharply with those few publications that convey a clear scientific approach on this topic. A scientist, not being an expert in the field, might easily conclude that adequate data to warrant the use of perforator-based reconstructions are too scarce, but every expert in the field of reconstructive surgery who has experience with perforator-based flaps would almost certainly contradict this conclusion. So, the real significance of this review might therefore not be in the answers obtained but in the question that remained: “how can we start improving the scientific foundation to choose the best treatment strategies.” There is certainly a need for high-quality studies to warrant the use of new (and old) treatment strategies. This would certainly improve our decision-making as well as the quality of treatment algorithms.

How do we interpret the findings in the included studies?

In this review, only eleven studies finally qualified following the search strategy that we presented. It has to be emphasized that we deliberately used low thresholds to consider studies for inclusion because of the anticipated low numbers of eligible studies. Lowering the threshold, any further would certainly lead to more inclusions but not to better founded conclusions.

However, despite our concerns on the level of evidence in general, the available studies are certainly not without meaning: firstly, perforator-based procedures appear to be as safe as free and local flaps, although only a few studies accurately measured the percentage of necrosis [8, 14]. The outcome of other studies, which were mostly retrospective and descriptive of nature, coincides with this conclusion. A practical issue of local flaps is the limited length-to-width ratio. Moreover, the availability is limited in extensively burned patients. For free flaps, more donor sites can be considered such as the anterolateral thigh perforator flaps, tensor fascia lata perforator flaps, and the thoracodorsal artery perforator flap [17, 20]. But local flaps procedures are generally considered less technical demand and require less operation time. They are also considered to be safer than free flaps. Nevertheless, Tsai et al. showed in their study that the application of free flaps for burn scar contracture could be safe. They described that all flaps healed well (only two re-explorations were necessary, which were successful).

The other 10 studies focused on local flaps. Generally, local perforator-based flaps are designed as island flaps, also known as propeller flaps, where the flap is released completely except for its vascular bundle with its cuff. After the inset, the skin pedicle looks like an island of skin. This concept was popularized by Hyakosuku since 1991 [6]. Cutting the entire skin base indeed provides large degrees of freedom to rotate the flap. However, in many cases, the flap only has to be rotated over a small angle (approximately 90°). In these cases, the flap is easily rotated with an intact base. These types of flaps are less surgically challenging, but theoretically, they also leave fewer scars, require less operation time, and are potentially safer. Mehrotra presented this idea as the perforator-plus design in 2007 [21]. In this approach seemingly, a conventional fasciocutaneous flap was created but the perforators were included at the base of the flap. This concept was considered to offer dual blood supply to the flap from the dissected perforator plus the flap base. In case series, it was found to be a valuable and safe technique [13, 21]. Our group developed an algorithm in which flaps were only converted into island flaps on indication such as significant kinking, compromised tissue perfusion, and dog-ear formation at the flap base [14]. The design of the flap was tailored according to the localization of an ad hoc perforator and the anticipated defect. The skin base was left intact until the flap rotation could be tested intraoperatively. This algorithm was versatile and practical because it allowed for tailoring intraoperatively.

Although we were convinced that local flaps are superior to skin grafting procedures for burn scar contracture treatment, we initiated an RCT comparing these perforator-based interposition flaps to full-thickness grafting [8]. The superiority of perforator-based flaps was indeed confirmed: with regard to the safety of the perforator-based interposition flaps, we found a relatively low percentage of necrosis compared to FTSGs (6% versus 17%). In all cases, a perforator was found adjacent to the contracture. In this RCT, we were able to demonstrate an even larger increase in surface area after 3- and 12-month follow-up. The flaps showed a 23% increase of the original surface area after 3 months and 42% after 1 year. On the other hand, a substantial contraction of the surface area was found for FTSGs after 3 months (−13%) and after 1 year (−8%). This is not only a statistically significant finding but also relevant for clinical decision-making. Although it was the first time that this difference in adaptation between a flap and FTSGs was demonstrated in a controlled study, it was not the first study where the increase of the flap size in time was observed. Tsai et al. and Rashid et al. objectified the increase of the flap width [12, 17]. In addition, Verhaegen et al. measured an increase in the flap surface area [14]. Waterston et al. did not perform geometry on flaps, but they observed and described this phenomenon in their case study [18].

This feature of flaps to stretch after interpositioning in an area of a released contracted burn scar is obviously a valuable practical advantage of perforator-based flaps in burn scar reconstruction. In flaps (local or free flaps), the quality of the tissue remains almost optimal after surgery probably because its vascularization remains intact instead of skin grafts, which are literally cut from their blood supply for days. Skin grafts can tolerate an ischemic period without being subject to necrosis; however, this is not favorable for the quality of the grafted skin [1].

In flaps, not only the vascularization is maintained but also the subdermal fat tissue and subdermal plexus. An important property of the subdermal fat tissue is to provide a functional sliding layer. The additional subcutaneous sliding layer of a flap prevents the skin from being attached to the underlying wound bed and allows the skin of the flap to stretch in time.

Two of the selected studies described the use of tissue expanders in combination with perforator-based flaps [10, 15]. Wang concluded that pre-expansion might facilitate the use of larger flaps and less problematic direct closure of the donor site. Perforator-based flaps can also be raised on scarred tissue as long as vascularization remains by the perforators and subdermal plexus remains intact [14, 18, 22]. This may be useful in scarred areas where not a sufficient amount of healthy skin is available. Supple scar tissue may be included in the flap design as well.

The discovery of perforators and the development of perforator-based flaps have changed the armamentarium of the reconstructive surgeon considerably. Maybe this development was so self-evident that there was no incentive for a more scientific approach to prove its effectiveness. This review taught us that the level of evidence to support the use of perforator-based procedures for burn scar contracture treatment is still poor. The most valuable information came from the studies with well-defined protocols, clear follow-up time, and using validated outcome measurement tools [8, 12, 14, 15, 17, 22]. There is a clear need to perform more studies of high quality. This is elegantly demonstrated by all four studies that critically evaluated flap size and width over time: they came with the salient finding that flap size and width increase over time. The question rises what happened with flap size in the other case series. Probably, flap adaption was present there as well but since it was not routinely measured, the authors probably missed this clinically relevant finding (although it was described in one study as an observation) [18]. The answer clearly remains elusive but it is certainly worthwhile to consider geometry for future studies.

#### Future perspectives

As disappointing as the evidence demonstrated by this review may be, it uncovers the flaws in the current available scientific literature and thereby provides important lessons for future studies investigating the effectiveness of burn scar contracture release surgery. First, a sufficient sample size should be chosen. A realistic power calculation is needed to determine the number of patients that should be acquired in a trial, depending on the effect of an intervention in a specific patient population. Second, the study should be designed in such a way that a comparison is made with another intervention. Third, we would like to stress the importance of the outcome assessment; it should be carefully linked to a relevant clinically expected outcome. We advocate the use of reliable and valid measurement techniques to assess the outcome, which allows for comparison between study results [23]. The introduction of new measurement tools, without validating them, is not recommended [23]. Finally, an adequate data presentation and statistical analysis are a necessity. Although RCTs are promoted as the holy grail for comparison of different treatment modalities, they have some shortcomings in terms of recruitment, ethics, patient preferences, and treatment comparisons [24]. Due to these shortcomings, the duration of RCTs is often long, which may hinder an adequate response to new developments. The ability to scientifically anticipate to new developments is essential for more evidence-based medicine in reconstructive surgery. Recently, a new design to overcome most of these shortcomings has been introduced: the cohort multiple randomized controlled trial (cmRCT) [24, 25]. The basis of the cmRCT is a large observational cohort of patients that is recruited for multiple trials in which a random selection of some participants is used for comparison between (new developed) interventions. This study design could allow for multiple comparisons simultaneously and a lower drop off due to patients’ preferences. We believe this could be a valuable study design in the field of reconstructive surgery where new techniques or adaptions to yet existing techniques are rapidly developing [26].

## Conclusions

To conclude, perforator-based interposition flaps appear to be highly relevant for burn scar contracture treatment but the evidence to support this treatment is poor. It is time to make steps forward to a more evidence-based medicine approach in reconstructive surgery. Implementation of the lessons that were addressed above should be pursued in future studies. We suggest repeating a review of literature with respect to this subject within a 5-year period to detect a shift towards better studies that enable a more evidence-based clinical practice within the burn field.

## Additional files

Additional file 1: Figure S1. The Newcastle-Ottawa quality assessment scale. (DOCX 80 kb)
